# Mycotic Abdominal Aortic Aneurysm Caused by Streptococcus equi

**DOI:** 10.7759/cureus.13899

**Published:** 2021-03-15

**Authors:** Danielle Schwartz, Donald McCarville, Alexander Wong

**Affiliations:** 1 Department of Medicine, University of Saskatchewan, Saskatoon, CAN; 2 Department of Vascular Surgery, University of Saskatchewan, Regina, CAN; 3 Department of Infectious Diseases, University of Saskatchewan, Regina, CAN

**Keywords:** mycotic aneurysm, streptococcus equi, abdominal aortic aneurysm

## Abstract

*Streptococcus equi* is a bacterium common in equine species and an uncommon pathogen in humans. Reported human infections can be severe and include meningitis, septic arthritis, and endocarditis. We report the case of a 64-year-old male who *S. equi *with several months of constitutional symptoms, back pain, and abdominal pain. Imaging demonstrated a large abdominal aortic aneurysm with a contained retroperitoneal rupture, with cultures from the aneurysm and blood cultures both positive for *S. equi*. The patient was successfully treated with open repair and placement of a Dacron graft and intravenous antibiotics and will remain on lifelong antibiotic prophylaxis.

## Introduction

Mycotic aneurysms are arterial dilations caused by pathogens weakening the vascular wall. They are uncommon, accounting for 0.6% of aortic aneurysms [1], and have a good long-term prognosis with surgical repair. Staphylococcus aureus, Salmonella species, and Streptococcus species are the most common causative organisms [2]. The recommended therapy for mycotic aneurysms is surgical repair and at least six weeks of antibiotic therapy.

*Streptococcus equi* is a zoonotic pathogen that rarely causes infection in humans. There are three subspecies, *S. equi* subsp. equi, *S. equi* subsp. ruminatorum, and *S. equi* subsp. zooepidemicus, of which *S. zooepidemicus* is the subspecies reported to cause disease in humans. *S. equi* is a β-hemolytic Lancefield group C streptococcal bacterium and is the causative bacterium in 1.4% of all group C streptococcal infections [3]. While it most commonly causes infections in horses, it also causes infections in other animals including cats, pigs, and goats [4-7]. It can be transmitted to humans via contact with an infected animal or from the ingestion of unpasteurized dairy products [8]. *S. equi* usually enters the body through the respiratory and gastrointestinal tract, as well as through skin wounds [9]. It is closely related to Streptococcus pyogenes [8] and commonly causes similar sequelae of infection in humans such as glomerulonephritis [10], as well as serious infections including meningitis, septic arthritis, endocarditis, pneumonia, and osteomyelitis [6,10-13]. Our report demonstrates that *S. equi*, a zoonotic Gram-positive coccus, can cause mycotic aneurysms, in addition to other invasive infections.

## Case presentation

A 64-year-old male presented to our facility with a three-month history of constitutional symptoms including generalized malaise, weight loss, anorexia, fatigue, and night sweats. Later in his course, but prior to presenting for medical care, he developed additional symptoms of back and abdominal pain. He was immunocompetent, and his medical history was significant for previous myocardial infarction, for which two drug-eluting stents were placed, and a perforated gastric ulcer repaired in 1990. He had no surgical history or family history of aortic aneurysms. His occupation was animal husbandry, and he worked on a farm that sells livestock with many cattle. He described an injury to the inside of his left leg after he slipped on ice and fell into cattle manure several months prior to the onset of his illness. It was a small open wound that was not reported to have clinical signs of infection; therefore, he did not receive antibiotics at that time. He did not smoke and denied past or current intravenous drug use.

As an outpatient, the patient previously underwent esophagogastroduodenoscopy to investigate his six-week history of abdominal pain and was found to have a gastric ulcer positive for Helicobacter organisms. He was treated with triple therapy of clarithromycin, amoxicillin, and pantoprazole. All of his symptoms improved while on this regimen, but they relapsed thereafter.

**Figure 1:**
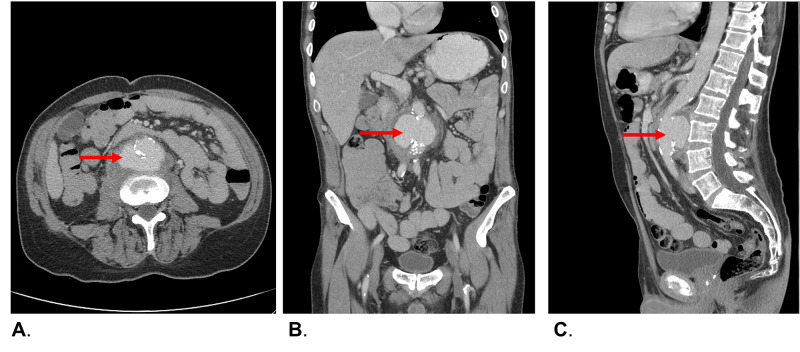
(A) Axial view demonstrating circumferential calcification of the infrarenal abdominal aorta, with normal appearing diameter of aortic lumen, with associated posterior pseudoaneurysm. (B) Coronal view of infrarenal abdominal aorta with large posterior pseudoaneurysm. (C) Sagittal view, posterior pseudoaneurysm, with preserved aortic diameter above and below the arterial defect. Note the compression of the aortic lumen superior to the pseudoaneurysm.

Given his abdominal and back pain, a non-urgent computed tomography (CT) scan was performed three months after his symptoms developed, which demonstrated a large aneurysm in the infrarenal abdominal aorta measuring 8.2 x 5.8 x 8.5 cm, with retroperitoneal fat stranding surrounding the aneurysm (Figures 1A-1C). He had never had any previous imaging of his abdomen for comparison. The patient was immediately transferred to vascular surgery at our center for assessment and definitive management.

On initial presentation to our facility, after the CT scan, the patient’s physical exam demonstrated a tender pulsatile abdominal mass. He was afebrile and vital signs were within normal limits. Peripheral pulses were within normal limits. There were no clinical manifestations of infective endocarditis. Laboratory investigations showed white blood cell count 10.3 x 109/L, hemoglobin 94 g/L, platelets 433 x 109/L, with elevated inflammatory markers (ESR 105 mm/hr, CRP 95.6 mg/L). The patient was taken immediately to the operating room for repair of what appeared to be a contained ruptured abdominal aortic aneurysm. Initial intra-operative intravenous antibiotics consisted of 2 g of cefazolin. Operative findings consisted of a small amount of ascites and a large abdominal aortic aneurysm with peri-aneurysmal edema, but without the expected peri-aneurysmal hematoma. After control and clamping of the vessels and upon opening the aorta, total loss of the posterior wall of the vessel was discovered, with a contained retroperitoneal rupture. The infected tissues were debrided and repair was completed with a synthetic Dacron prosthesis. Intra-operative samples were submitted including swabs of the presumed infected material as well as a piece of thrombus. Given the suspected mycotic nature of the aneurysm, post-operative antibiotics were broadened to piperacillin/tazobactam and vancomycin.

All intra-operative cultures as well as post-operative blood cultures were positive for *S. equi*, susceptible to penicillin (MIC ≤ 0.06). The infectious disease service transitioned antibiotics to ceftriaxone 2 g IV q24h. Blood cultures drawn 96 hours post-operatively were sterile, and the patient’s clinical condition improved rapidly. Following an unremarkable seven-day post-operative course, the patient was discharged home with a six-week course of ceftriaxone from the day of blood culture sterilization.

At the completion of his course of intravenous antibiotics, the patient reported near-complete resolution of symptoms with reassuring laboratory investigations. White blood cell count was 4.7 x 109/L, hemoglobin 100 g/L, platelets 323 x 109/L, and inflammatory markers were trending towards normal (ESR 64 mm/hr, CRP 14.4 mg/L). Intravenous antibiotics were discontinued, and he began amoxicillin 500 mg orally three times daily for six weeks. At review following 12 weeks of cumulative antibiotic therapy, he had returned to his previous functional baseline with no complaints of note, and laboratory investigations had normalized completely. He was switched to amoxicillin 500 mg orally twice daily for lifelong prophylaxis.

A repeat CT scan of the aorta was performed four months after surgery, which showed mildly ectatic changes consisting of the Dacron prosthesis and peri-aortic tissues measuring 3.1 x 3.9 cm. No anostamotic pseudo-aneurysms were seen. A small intra-luminal linear density was noted but consisted of a localized fold in the Dacron graft, which is clinically insignificant.

## Discussion

The patients with mycotic aneurysms tend to present with pain, fever, elevated inflammatory markers, leukocytosis and bacteremia, and many aneurysms are found ruptured when patients are taken for surgery [14,15]. Our patient presented with the majority of these symptoms but with insidious onset and months of non-specific symptoms, and incidental diagnosis following non-urgent imaging. The causative organism was found to be *S. equi*, which is not a typical organism that causes mycotic aneurysms. In our case, the subspecies of *S. equi* was not identified. The patient’s risk factors were working on a farm with animals and his skin wound.

Our patient was treated with guideline-recommended therapy for mycotic aneurysms, which is surgical repair and at least six weeks of antibiotic therapy. Surgical approaches include in-situ repair with synthetic prosthesis such as with our patient, in-situ repair with autogenous tissue, or ligation and extra-anatomic bypass. The duration of antibiotic treatment ranges from six weeks to lifelong prophylaxis, depending on the causative organism and aneurysm location [16]. Aortic graft infections occur in 1%-5% of cases and are associated with significant morbidity and mortality, with a mortality rate of about 19% [17], and *S. equi* often causes severe infections. Therefore, our intent is for the patient to remain on lifelong antibiotic prophylaxis. *S. equi* has been reported to cause secondary infections of atherosclerotic abdominal aorta aneurysms and vascular grafts [18,19]. Our report demonstrates that the organism can cause mycotic aneurysms as well.

## Conclusions

*S. equi *is an uncommon zoonotic bacterium that can cause a wide range of infections, including mycotic aneurysms. It should be considered when a patient presents with appropriate risk factors such as working closely with animals. Aneurysms caused by *S. equi* can be successfully treated in the same way as other bacterial mycotic aneurysms, with surgery to repair the aneurysm and prolonged antibiotic therapy, and lifelong prophylaxis in selected candidates.
